# Public Health and Rare Diseases: Oxymoron No More

**DOI:** 10.5888/pcd13.150491

**Published:** 2016-01-14

**Authors:** Rodolfo Valdez, Lijing Ouyang, Julie Bolen

**Affiliations:** Author Affiliations: Lijing Ouyang, Julie Bolen, National Center on Birth Defects and Developmental Disabilities, Centers for Disease Control and Prevention, Atlanta, Georgia.

The mission of public health has been succinctly stated as “the fulfillment of society’s interest in assuring conditions in which people can be healthy” (1). Public and private institutions charged with this mission monitor population health and respond when threats arise. These may be sudden health crises (eg, infectious outbreaks), persistent health problems (eg, chronic diseases), or buildups of environmental risk factors (eg, pollution). Public health practitioners use a combination of disciplines that include basic science, clinical research, epidemiology, statistics, behavioral research, health care services, economics, and policy to identify the primary or secondary causes of health threats and then systematically prevent, mitigate, or suppress these causes in entire populations. This approach has been tremendously successful against infectious diseases and has had notable successes against injuries, accidents, and major chronic diseases (2,3). The purpose of this essay is to highlight the case of a group of nearly 7,000 rare (low-prevalence) diseases, mostly of genetic or congenital origin, for which the applicability of the public health approach, primary prevention in particular, seems limited. We argue that a wider application of this approach could greatly benefit the patients affected by these diseases and their relatives. We start by presenting the challenges of implementing this approach for rare diseases, then we present the need for such an approach and a few notable examples of its successful application to these diseases. Finally, we provide a structured list of public health activities that are key to the management of rare diseases in populations. 

A rare disease is a condition that affects fewer than 200,000 people in the United States or no more than 1 of every 2,000 people in Europe (4). Examples of rare diseases are life-threatening and physically or mentally disabling conditions such as Huntington disease, spina bifida, fragile X syndrome, Guillain-Barré syndrome, Crohn disease, cystic fibrosis, and Duchenne muscular dystrophy. Possibly the main reason for the limited applicability of the public health approach to rare diseases is that patients are few and scattered across populations. But another reason is that approaches based on identifying and removing risk factors are generally not well-suited for diseases whose primary risk factors are innate or congenital and therefore irremovable.

Additionally, many rare diseases have a long list of characteristics that present serious challenges for public health practitioners (5). Among rare diseases it is common to find that 1) diagnoses are difficult and delayed; 2) case definitions for surveillance are usually lacking; 3) International Classification of Diseases (ICD) codes for record keeping are poorly defined or not assigned; 4) underlying molecular or physiologic mechanisms are unknown; 5) specialized and coordinated medical care is in short supply, and treatments can be complex; 6) standards of care for treatment and rehabilitation are not evidence-based because health research is necessarily done at small scale; 7) longitudinal data collections are scarce; 8) the development of new medications and treatments can be fragmented and slow; 9) screening strategies lack efficiency; and 10) scope and capacity of most registries and databases are limited. Our knowledge of most rare diseases is so insufficient that they are also known as orphan diseases because of their failure to attract the interest of researchers, medical specialists, drug makers, and policy makers (5).

Despite the challenges, we have compelling reasons to apply a public health approach to rare diseases: they collectively affect about 25 million people in the United States, about 30 million in Europe, and about 400 million worldwide (4); most rare diseases begin in childhood and can have devastating health consequences, including premature death. They can severely affect the lives of caregivers; their economic impact is often substantial for patients, their families, and society in general (6,7). Although rare diseases are a common cause of neurological and intellectual disabilities and many have no cure, some can be prevented or controlled and the lifespan of patients can be extended into adulthood with opportune medical interventions. A few large-scale public health approaches that have been successfully used on rare diseases offer concrete examples in favor of this view. For decades, screening of newborns has been used to ameliorate or prevent adverse metabolic and developmental consequences among children born with treatable rare conditions in the United States and other countries (8,9). Mandatory folic acid fortification of enriched cereal grain products has contributed to the reduction of neural tube defects, including spina bifida, in populations (10,11). The life expectancy of cystic fibrosis patients has increased from under 10 to over 40 years of age in the past few decades as advances in medical care reach larger segments of their population (12). The prevalence of a severe genetic disease, Tay-Sachs, has been drastically reduced among the Ashkenazi Jewish population through population screening and strategies such as prenatal diagnosis for carrier couples and marriage avoidance between carriers (13,14).

Important support for a public health approach to rare diseases has also come through the services rendered by patient organizations to their members and through policy efforts that include the passage of legislation such as the Orphan Drug Act and the Rare Diseases Act, which have encouraged research and drug development. Evidence suggests that efforts of this type have empowered patients with rare diseases and their organizations as they seek and obtain wider social recognition, more participation in research, and better health care (15,16).

Given the difficulty of the challenges involved, public health approaches may not seem suitable for rare diseases when the primary measures of success relate to the prevention of large numbers of cases or the avoidance of premature deaths. A closer look, however, may confirm that individuals affected by disabling, life-threatening, and largely unpreventable diseases benefit enormously from a comprehensive public health approach to control conditions associated with these diseases in their populations. Although we want to keep focus on primary prevention whenever possible, an alternative measure of success on rare diseases could be, for example, slowing down the clinical progression while reducing their adverse impact on the lives of affected individuals, their relatives, and their caregivers. A host of activities that can be undertaken to control rare diseases fall within the domain of public health. The Box lists goals for a comprehensive public health approach to rare diseases. Generally, these activities have been proposed for single rare diseases, but much more can be gained from a public health standpoint by focusing these actions and measuring their impact on groups of rare diseases with common characteristics and risk factors.

**Box. Goals for a Comprehensive Public Health Approach to Rare Diseases and Its Potential Impact on Affected Populations**

**Impact of disease**

- Define the population impact through epidemiological research adequate for low-prevalence diseases
- Examine costs associated with these diseases, such as medical costs, lifetime productivity loss, financial impact on caregivers, and impact on the employability of patients

**Surveillance**

- Develop and implement wide-ranging registries and surveillance systems
- Establish common screening and proper diagnostic procedures
- Monitor trends and the distribution of the diseases across races and geographic areas
- Develop case definitions and ICD codes to facilitate record keeping and surveillance through large administrative data sets

**Knowledge**

- Improve the methods and timing of diagnosis
- Develop clinical practice guidelines and quality measures to facilitate their evaluation
- Develop or apply new statistical methods for clinical trials involving diseases with low prevalence
- Describe clinical courses of diseases and their associated health conditions
- Document progression of health status and associated quality of life
- Evaluate health outcomes (both cross-sectional and longitudinal)
- Facilitate access to promising experimental treatments (clinical trials)
- Identify research priorities and new therapeutic advances
- Create regional or national networks to share research knowledge and clinical expertise
- Use current or create large national or regional databases from, for example, Medicaid and all payer claims to allow the study of substantial numbers of patients

**Health care**

- Concentrate health care efforts on aspects of the diseases that can be prevented or alleviated
- Evaluate and compare health care practices
- Identify and disseminate evidence-based findings on best practices and standards of care
- Encourage pharmaceutical companies and device makers to develop new treatments and tools
- Develop case definitions and ICD codes to facilitate reimbursement
- Consider the development of regional centers of clinical expertise that would make specialty care widely available to patients
- Monitor the performance of existing therapies
- Consider the expansion of specialized social and community services
- Guide the planning and implementation of treatments and interventions
- Create regional networks of health care providers within which treatment and reimbursement are coordinated
- Establish viable options for access to proper care regardless of place of residence (eg, telemedicine)

**Potential benefits**

- A reduced impact of disease on patients, their relatives and caregivers, and society in general
- Favorable changes in disability and mortality figures
- Better management of associated health conditions
- A smooth transition between pediatric and adult care
- Improved health, quality of life, and life expectancy
- Enhanced participation of patients in their communities, workplaces, and society in general

Note: Some of the goals listed here can be found in the websites or documents of several organizations devoted to rare diseases, namely, Orphanet (information on rare diseases and orphan drugs): www.orpha.net; Rare Diseases Europe, www.eurordis.org; The National Organization for Rare Disorders, www.rarediseases.org; and the Australian Paediatric Surveillance Unit, www.apsu.org.au.
