# Erratum, Vol. 13, January 14 Release

**DOI:** 10.5888/pcd13.150491e

**Published:** 2016-02-11

In the article “Public Health and Rare Diseases: Oxymoron No More,” boxed text was omitted because of a technical error.

The correction was made to our website on January 15, 2016, and appears online at http://www.cdc.gov/pcd/issues/2016/15_0491.htm. We regret any confusion or inconvenience this error may have caused.

